# Protein Lipidation by Palmitate Controls Macrophage Function

**DOI:** 10.3390/cells11030565

**Published:** 2022-02-06

**Authors:** Jeroen Guns, Sam Vanherle, Jerome J. A. Hendriks, Jeroen F. J. Bogie

**Affiliations:** 1 Department of Immunology and Infection, Biomedical Research Institute, Hasselt University, 3590 Diepenbeek, Belgium; jeroen.guns@student.uhasselt.be (J.G.); sam.vanherle@uhasselt.be (S.V.); jerome.hendriks@uhasselt.be (J.J.A.H.); 2 University MS Center, Hasselt University, 3500 Hasselt, Belgium

**Keywords:** protein acetylation, innate immunity, macrophages, S-palmitoylation, inflammation

## Abstract

Macrophages are present in all tissues within our body, where they promote tissue homeostasis by responding to microenvironmental triggers, not only through clearance of pathogens and apoptotic cells but also via trophic, regulatory, and repair functions. To accomplish these divergent functions, tremendous dynamic fine-tuning of their physiology is needed. Emerging evidence indicates that S-palmitoylation, a reversible post-translational modification that involves the linkage of the saturated fatty acid palmitate to protein cysteine residues, directs many aspects of macrophage physiology in health and disease. By controlling protein activity, stability, trafficking, and protein–protein interactions, studies identified a key role of S-palmitoylation in endocytosis, inflammatory signaling, chemotaxis, and lysosomal function. Here, we provide an in-depth overview of the impact of S-palmitoylation on these cellular processes in macrophages in health and disease. Findings discussed in this review highlight the therapeutic potential of modulators of S-palmitoylation in immunopathologies, ranging from infectious and chronic inflammatory disorders to metabolic conditions.

## 1. Protein Acetylation

Cellular proteins undergo numerous post-translational modifications that extend and regulate protein and, thereby, cellular physiology. Some of these modifications, phosphorylation, and ubiquitination, in particular, have been extensively studied and characterized in the context of cellular function in health and disease [1]. In contrast, only recently, owing to technological advances allowing their accurate measurement, we are beginning to understand the essential role of acetylation on protein physiology and cellular function.

Depending on the nature of the fatty acids attached to a protein, acetylation can take multiple forms, the three most commonly known being prenylation, N-myristoylation, and palmitoylation [2,3,4]. Protein prenylation catalyzes the covalent attachment of either a 15-carbon (farnesyl) or 20-carbon (geranylgeranyl) isoprenoid lipid to free thiols or cysteine side-chains at or near the C-terminus of proteins. Emerging evidence stresses the importance of this form of acetylation in driving cellular activity, trafficking, and localization of RAS family GTPases and heterotrimeric G-proteins [5,6]. N-myristoylation is characterized by the addition of myristic acid, a 14-carbon unsaturated fatty acid, to the alpha-amino group of an N-terminal glycine residue of a wide range of substrate proteins. It plays a vital role in signal transduction, protein stability, and the localization of proteins to membranes [7,8]. Recent studies further indicate that N-myristoylation is involved in host defense against microbial and viral infections (reviewed in [9]). Protein palmitoylation involves the covalent binding of palmitate to amino acid residues of proteins [10] and can be subdivided into S- and N- palmitoylation. S-palmitoylation involves the addition of palmitate to cysteine sulfhydryl groups of proteins via a labile thioester linkage. Ample evidence indicates that S-palmitoylation plays an important role in protein trafficking, targeting, and stability and tightly controls protein–protein interactions [11,12]. N-palmitoylation is a less common form of palmitoylation and involves the binding of palmitate to amide groups of lysines, glycines, and N-terminal portions of proteins. In contrast to S-palmitoylation, N-palmitoylation is an irreversible and poorly understood process. However, few studies support the role of N-palmitoylation in protein function and localization [12,13]. In this review, we summarize and discuss the current knowledge on the impact of S-palmitoylation, one of the most common forms of protein lipidation, on protein signaling, translocation, and interactions in innate immune cells, macrophages in particular.

## 2. S-Palmitoylation

**Figure 1:**
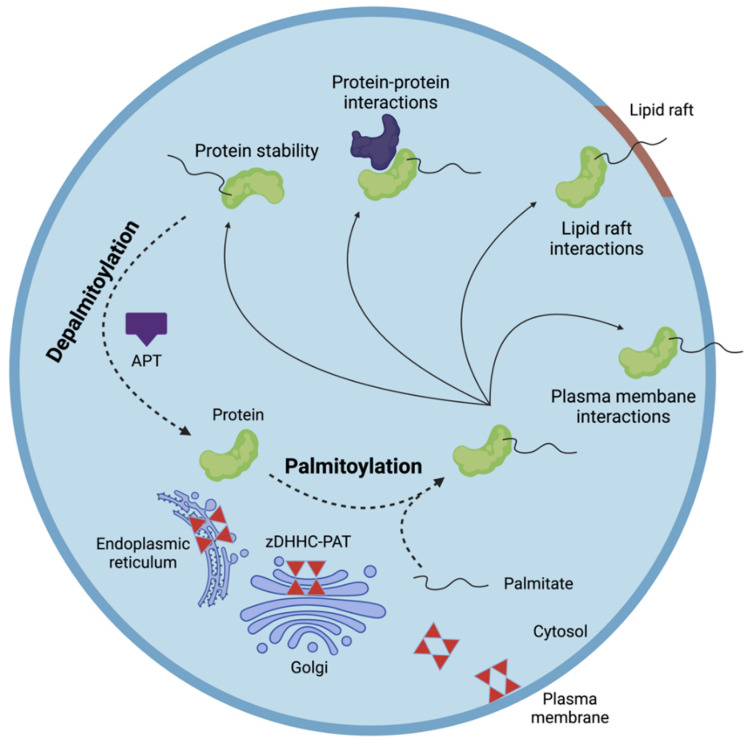
Dynamic protein S-palmitoylation. S-palmitoylation is a reversible and dynamic post-translational protein modification that is mediated by protein acyl-transferases (zDHHC-PAT) and acyl protein thioesterases (APTs) that attach to and remove palmitate from protein cysteine residues, respectively. S-palmitoylation controls protein activity, stability, trafficking, and protein–protein interactions. Figure created with BioRender.com (accessed on 9 January 2022).

The saturated fatty acid palmitate (C16:0) is the most common fatty acid in the human body. It is situated at the heart of fatty acid metabolism, as it is the primary end-product of fatty acid synthesis and represents the major substrate for cellular β-oxidation and desaturation [14]. As shortly mentioned above, S-palmitoylation is a post-translational modification that consists of the reversible addition of palmitate to cysteine residues on proteins through a thioester bond [15,16]. Upon S-palmitoylation, the hydrophobicity of proteins increases, allowing proteins to associate with membranes of intracellular organelles, such as the endoplasmic reticulum, Golgi apparatus, endosomes, mitochondria, and the plasma membrane. Of interest, few studies demonstrated that S-palmitoylation can impact the activity of transcription factors as well. Noland et al. demonstrated that S-palmitoylation controls folding and stability of TEAD family transcription factors [17,18], which are involved in cell growth and differentiation. Likewise, a mutated form of the small GTPase Cdc42 is linked with increased S-palmitoylation and associated with enhanced activation of the NF-κβ transcription factor and signaling pathway [19]. All in all, S-palmitoylation dynamically regulates protein life cycle and function by facilitating membrane interactions and trafficking and by modulating protein–protein interactions and enzyme activity (Figure 1) [20,21,22,23].

Palmitoyl-proteome screens and prediction algorithms indicate that at least 10% of the human proteome is susceptible to S-palmitoylation. Therefore, S-palmitoylation is considered to be among the most prevalent post-translation lipid modifications [24]. In general, most post-translational lipid modifications are static and rely on specific substrate sequence motifs [25]. However, unlike analogous lipid modifications, such as N-myristoylation and prenylation, S-palmitoylation is an enzymatically reversible process and lacks a specific sequence motif [15,23]. These characteristics stress the dynamic nature of S-palmitoylation and have hampered the prediction of substrate targets of S-palmitoylation for a long time. Despite the latter, recent studies showed that the addition of palmitate typically occurs on cysteine residues in the vicinity or within transmembrane domains or near existing membrane-targeting protein-lipid modifications, such as prenylated cysteine or N-terminal myristoylated glycine residues [15,23].

While S-palmitoylation can occur spontaneously, auto-palmitoylation, it is assumed to be primarily driven by protein acyl-transferases with zinc-finger and aspartate-histidine-histidine-cysteine (zDHHC) domains [26,27]. These zDHHCs catalyze S-palmitoylation via a two-step process, in which they are first auto-palmitoylated to form a palmitoyl-zDHHC complex followed by transfer of palmitoyl to substrate proteins [28]. The reverse process, de-palmitoylation, in which palmitate is removed from proteins, is mediated by acylprotein thioesterases (APTs) (Figure 1). The zDHHC family of enzymes consists of 24 proteins in humans (zDHHC1-24), while at least six APTs are described, including palmitoyl-proteinthioesterase-1/-2, APT1/2, and α/β hydrolase domain-10/-17 [22,25,26,29,30]. Interestingly, our unpublished findings indicate that zDHHCs and APTs have divergent cellular and tissular distributions, and few studies showed specific subcellular locations of these enzymes [13,22,31,32]. With respect to the latter, the majority of zDHHCs are localized in both the endoplasmic reticulum and Golgi apparatus, which are the primary sites of protein palmitoylation. While zDHHC7 and zDHHC8 are specific to the Golgi apparatus, zDHHC6 and zDHHC13 are specific to the endoplasmic reticulum. On the other hand, zDHHC5, zDHHC20, and zDHHC21 are primarily localized at the plasma membrane [13,22]. In contrast, APTs are mainly situated in the cytosol but are, in small proportions, also present in the plasma and nuclear membrane, as well as the endoplasmic reticulum [31,32]. In addition to context-dependent changes in expression and differences in cellular distribution, cellular localization of zDHHCs is likely to drive substrate specificity of zDHHCs.

Various methods have been developed to determine protein palmitoylation dynamics. For a long time, metabolic radioactive labeling using 3H-, 14C- or 125I-labeled palmitic acid was the most commonly used technique to detect S-palmitoylation in live cells. However, given the time-consuming and laborious nature as well as lack of sensitivity and specificity, acyl-biotin exchange (ABE) and acyl-resin-assisted capture (Acyl-RAC) replaced radiolabeling techniques (reviewed in [33]). The principle of ABE originates from the labile nature of thioester bonds between palmitate and cysteine residues. Upon blocking free cysteine residues, neutral hydroxylamine is used to cleave the labile thioester bonds between palmitate and palmitoylated cysteine residues, after which newly revealed cysteines can be labeled and captured using a sulfhydryl-reactive biotinylation reagent and streptavidin beads, respectively. Acyl-RAC follows largely the same experimental pipeline. However, exposed cysteine residues are immediately conjugated to thiopropyl sepharose-containing beads. Finally, click chemistry has advanced the field of protein palmitoylation by providing specific, sensitive, rapid, and easy-to-handle methods for studying protein palmitoylation (reviewed in [34]). Here, azido- or alkynyl-containing fatty acids, commonly 17-octadecyonic acid (17-ODYA) for S-palmitoylation, are metabolized by the endogenous cellular palmitoylation machinery and transferred to endogenous sites of modification. The azide or alkyne group can be conjugated to reporter molecules, such as fluorophores or biotin derivatives, allowing for targeted and untargeted identification of palmitoylated proteins. For all the above mentioned techniques, targeted immunoblotting and unbiased mass spectrometry-based proteomics techniques are generally applied to identify palmitoylated proteins.

## 3. S-Palmitoylation in Macrophages

Macrophages are multifunctional innate immune cells situated in all tissues within the body. While they are well-known for their essential role in the clearance of pathogens and controlling inflammatory responses, their functions go well beyond immunity. For instance, studies show that they are also indispensable for lipid metabolism as well as tissue development and remodeling [35]. To accomplish these divergent functions, tremendous fine-tuning of their physiology is required. Increasing evidence indicates that S-palmitoylation is key in driving appropriate responses of macrophages in processes such as endocytosis, intracellular signaling pathways, lysosomal hydrolase sorting, and chemotaxis. In the following sections, we summarize and discuss the current knowledge on the impact of S-palmitoylation on macrophage physiology in health and disease.

### 3.1. Endocytosis

The clearance of pathogens, cell debris, and apoptotic cells retains immune and tissue homeostasis in health and disease. Macrophages are equipped with an arsenal of receptors that recognize these ligands, many of which require S-palmitoylation for their sequestration in lipid rafts and function [15,36]. Moreover, few studies indicate that pathogens can hijack the palmitoylation machinery involved in the acetylation of some of these receptors to support their survival and spread.

**Figure 2:**
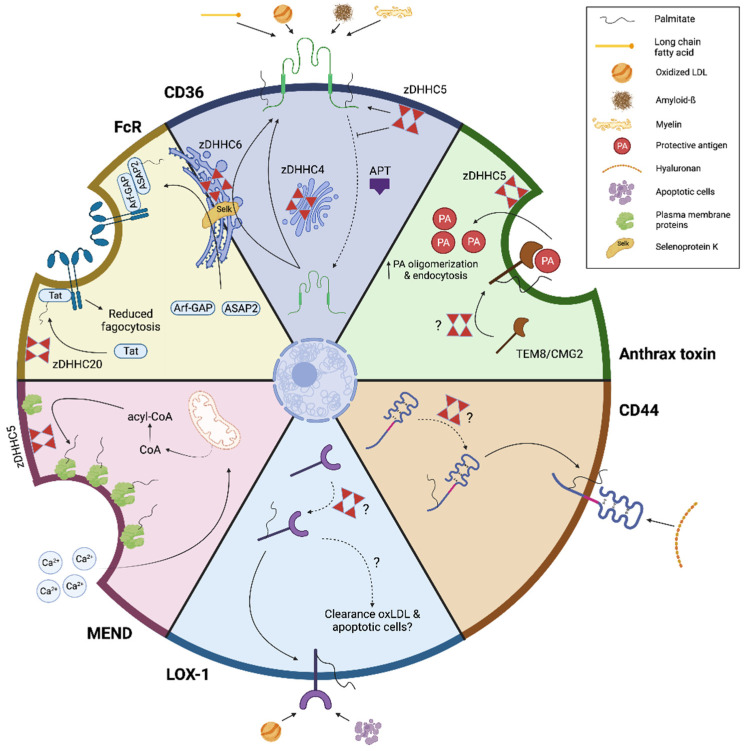
S-palmitoylation controls macrophage endocytosis. Ample evidence indicates that many endocytic receptors on macrophages require S-palmitoylation for their sequestration in lipid rafts and function. First, Fc receptor-mediated endocytosis relies on S-palmitoylation. Here, zDHHC6/SelK-mediated S-palmitoylation of the scaffolding protein ASAP2 was found to inhibit FC receptor-mediated endocytosis. Similar, zDHHC20-mediated S-palmitoylation of the viral toxin Tat was found to reduce FcR-mediated phagocytosis during HIV-1 infections. Second, the intracellular transport, plasma membrane localization, and function of the scavenger receptor CD36 depend on palmitoylation by zDHHC4–6 and Selenoprotein K (SelK). Third, the anthrax toxin produced by Bacillus anthracis hijacks the palmitoylation machinery to affect macrophage endocytosis. Here, S-palmitoylation of plasma membrane receptors TEM8 and CMG2, as well as zDHHC5-mediated S-palmitoylation of the proteases furin and PC7, was found to affect raft association, oligomerization, and endocytosis of protective antigen (PA), a subunit of anthrax toxin. Fourth, few studies indicate that the endocytic receptors CD44 and lectin-like oxLDL receptor-1 (LOX-1) rely on palmitoylation for proper plasma membrane distribution. The zDHHCs and acylprotein thioesterases (APTs), as well as the functional impact CD44 and LOX-1 S-palmitoylation, remain to be determined. Finally, S-palmitoylation triggers a process coined massive endocytosis (MEND) through zDHHC5-mediated S-palmitoylation of plasma membrane proteins in lipid rafts. Figure created with BioRender.com (accessed on 9 January 2022).

The first well-investigated scavenger receptor whose function is regulated by S-palmitoylation is the fatty acid translocase CD36. CD36 facilitates cellular uptake of long-chain fatty acids, a key step in energy metabolism [37]. Similar, macrophages recognize and ingest disease-associated ligands such as oxidized LDL (oxLDL), amyloid-β, and myelin via CD36, thereby having a major impact on disease pathogenesis in atherosclerosis, Alzheimer’s disease (AD), and multiple sclerosis (MS) [38,39]. Several studies found that CD36 is palmitoylated on multiple cysteine residues located in its N- and C-terminal cytoplasmic tail [40]. While S-palmitoylation of CD36 is redundant for receptor maturation, it controls endoplasmic reticulum processing, trafficking through the secretory pathway, and lipid raft localization [41]. Consistent with reduced lipid raft localization, CD36 S-palmitoylation mutants show less efficient uptake of oxLDL [41]. Selenoprotein K, an endoplasmic reticulum-localized protein that exhibits oxidoreductase enzymatic activity, was found to be an important co-factor in CD36 S-palmitoylation, directing its localization to lipid rafts in macrophages [42], potentially through its interaction with zDHHC6 [43,44]. Alternatively, or in parallel, CD36 S-palmitoylation, plasma membrane localization, and fatty acid uptake activity rely on zDHHC4 and zDHHC5 [45]. While zDHHC4 promotes CD36 S-palmitoylation in the Golgi apparatus and stimulates the transport of CD36 to the plasma membrane, zDHHC5 maintains plasma membrane localization of CD36 by protecting it from de-palmitoylation by APTs (Figure 2) [45]. Despite these studies, the impact of disturbances in CD36 S-palmitoylation on disease pathologies remains unexplored. We anticipate that changes in CD36 S-palmitoylation in macrophages will have disease-dependent outcomes. Given that CD36-mediated clearance of amyloid-β and myelin debris by macrophages is neuroprotective in AD and MS, respectively, CD36 S-palmitoylation may limit disease progression in these disorders. In contrast, excessive uptake of oxLDL and fatty acids by CD36 promotes atherosclerotic lesion development, inflammation, and liver steatosis. Hence, CD36 S-palmitoylation may promote disease progression in these disorders. Consistent with the latter, patients with non-alcoholic steatohepatitis exhibit elevated CD36 S-palmitoylation, which augments liver steatosis and inflammation [46]. Altogether, these findings indicate that -palmitoylation is essential for CD36 localization and function in macrophages. However, more research is warranted to define the importance of CD36 S-palmitoylation in disease pathology.

Antibody-opsonized pathogens and particles are recognized and engulfed by macrophages through Fc receptors (FcRs). FcR-mediated endocytosis is crucial for inducing appropriate responses to infections and chronic inflammation but can also contribute to the pathogenesis and progression of autoimmunity [47]. A recent study defined that palmitoylation indirectly facilitates FcR-mediated uptake of immunoglobulin G-coated microspheres by driving S-palmitoylation of Arf-GAP with SH3 domain, ANK repeat, and PH domain-containing protein 2 (ASAP2) [48], a scaffolding protein linked to FcR-mediated phagocytosis [49]. The zDHHC6-selenoprotein K complex was found to catalyze palmitoylation of ASAP2 at the endoplasmic reticulum, thereby directing its localization to phagocytic cups that appear beneath IgG-opsonized ligands. Counterintuitively, whereas S-palmitoylation generally stabilizes protein expression, selenoprotein K-mediated S-palmitoylation of ASAP2 marked it for calpain-2-mediated cleavage. It remains unclear how ASAP2 retention in phagocytic cups in palmitoylation-deficient cells leads to less efficient FcR-mediated phagocytosis [44]. In another study, S-palmitoylation was found to retain the viral toxin Tat, which is released by HIV-1 infected cells, in the plasma membrane of receiving uninfected macrophages, thereby inhibiting FcγR-mediated phagocytosis [50]. zDHHC20-mediated S-palmitoylation of Tat at Cys31 enabled Tat accumulation on phosphatidylinositol (4,5) bisphosphate (PI(4,5)P_2_) at the plasma membrane, and by doing so, allowing it to inhibit PI(4,5)P_2_-dependent phagocytosis (Figure 2). It is tempting to speculate that Tat impedes FcR-mediated phagocytosis by interfering with PI(4,5)P_2_-mediated recruitment of the Rho GTPases Cdc42 to the phagocytic cup [51]. These findings provide a molecular basis for the phagocytic defects observed in uninfected phagocytes following HIV-1 infection [52]. Collectively, these studies stress that S-palmitoylation is key in driving FcR function on macrophages.

Similar to HIV-1, virulence factors released by extracellular bacteria can hijack the endocytic capacity of cells to promote their survival and spread. A well-studied example is the anthrax toxin, one of the major virulence factors produced by *Bacillus anthracis*, which targets macrophages and neutrophils to successfully establish infection [53,54]. For anthrax toxin to enter macrophages, the protective antigen (PA) subunit of the toxin first binds to the plasma membrane receptors tumor endothelial marker 8 (TEM8) and capillary morphogenesis gene 2 (CMG2) [53,55]. Next, PA becomes cleaved by proteases such as furin and PC7, leading to PA oligomerization and complex endocytosis [56]. Several studies demonstrate that S-palmitoylation is essential for the entry of anthrax toxin in target cells. S-palmitoylation of TEM8 and CMG2 is found to affect PA raft association, oligomerization, and endocytosis, likely via a complex process involving other palmitoylated proteins [57]. A more recent study extends these findings by showing that zDHHC5-mediated S-palmitoylation of Furin and PC7 enables partitioning of these proteases into lipid rafts and is necessary for efficient PA oligomerization and endocytosis (Figure 2) [58]. Remarkably, similar processes control cellular intoxication, lipid raft localization, and oligomerization of aerolysin, a protoxin produced by *Aeromonas hydrophila* [58].

In addition to the abovementioned endocytic pathways, S-palmitoylation affects lipid raft localization and function of several other endocytic receptors expressed by macrophages. Alongside its role as an adhesion molecule, CD44 acts as a primary and accessory phagocytic receptor [59,60]. S-palmitoylation of CD44 is essential for its sequestration in lipid rafts and endocytosis of hyaluronan [61,62], a large inflammation-associated glycosaminoglycan that is cleared by macrophages in a CD44-dependent manner (Figure 2) [63]. Furthermore, S-palmitoylation of the scavenger receptor lectin-like oxLDL receptor-1 (LOX-1) at Cys36 and Cys46 is necessary for its recruitment into lipid rafts [64]. While direct evidence is lacking, LOX-1 S-palmitoylation may well impact the capacity of macrophages to clear oxLDL and apoptotic cells (Figure 2) [65]. Finally, S-palmitoylation triggers a process coined massive endocytosis (MEND), a form of endocytosis in which large parts of the plasma membrane are absorbed by cells, including macrophages [66,67]. MEND ensues following a large influx of calcium, which leads to the formation of mitochondrial transition pores, release of coenzyme A (CoA) in the cytoplasm, and formation of acyl-CoA. The latter serves as a substrate for zDHHC5-mediated S-palmitoylation of plasma membrane proteins, thereby increasing their clustering into ordered domains (Figure 2).

Collectively, S-palmitoylation is increasingly being acknowledged to impact the endocytic capacity of macrophages. However, we are only beginning to understand the molecular processes and receptors involved, let alone their impact on disease pathologies. By using the murine RAW264.7 macrophages cell line, palmitoyl screens identified numerous other palmitoylated candidates that are likely to affect the clearance of pathogens, cell debris, and apoptotic cells by macrophages, including but not limited to several members of the vesicle-associated membrane protein family [15,36]. In the upcoming years, more research is needed to confirm the impact of these putative palmitoylated proteins on the endocytic capacity of macrophages in health and disease.

### 3.2. Toll-like Receptor Signaling

Toll-like receptors (TLRs) are pattern recognition receptors that primarily sense conserved microbial ligands. Their ligation triggers the activation of transcription factors that control the expression of an array of inflammatory and anti-microbial genes. As immune sentinels, macrophages highly express TLRs, enabling them to rapidly respond to microbial threats [68]. TLR1, 2, 4, 5, and 6 are localized in the plasma membrane, where they recognize external microbial ligands. TLR3, 7, 8, and 9 are situated in cytoplasmic compartments and mainly recognize intracellular viral products.

**Figure 3:**
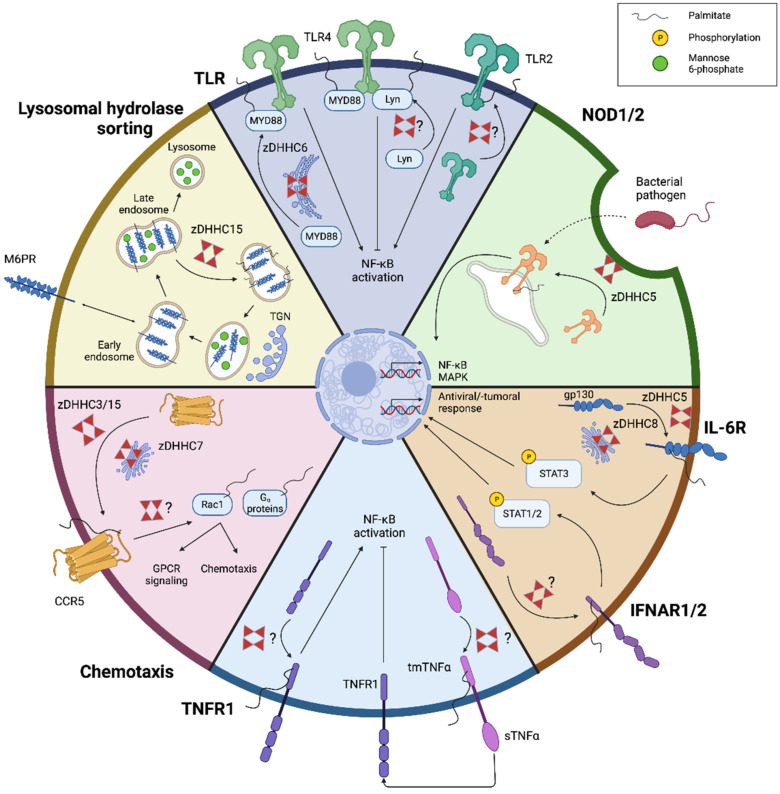
S-palmitoylation regulates lysosomal hydrolase sorting and cytokine, chemokine, NOD, and TLR signaling. First, few studies defined that S-palmitoylation controls TLR4 signaling by affecting the activity and binding properties of the adaptor molecule MYD88 and tyrosine kinase Lyn, thereby promoting or inhibiting TLR4 signal transduction, respectively. Moreover, TLR2 membrane distribution as such depends on S-palmitoylation. Second, membrane localization and ability to activate NF-κB and MAPK of NOD1/2 is associated with zDHHC5-mediated S-palmitoylation. Third, diverse cytokine and chemokine receptors expressed by macrophages rely on S-palmitoylation for signal transduction. For instance, zDHHC5/8-mediated palmitoylation of gp130, a subunit of the IL-6 receptor complex, affects its surface localization and downstream STAT3 phosphorylation. Likewise, S-palmitoylation of the interferon alpha and beta receptor subunits 1 and 2 (IFNAR1/2) controls its membrane abundance and downstream STAT1/2 phosphorylation. In addition, TNFα signaling is closely associated with S-palmitoylation in macrophages. S-palmitoylation of transmembrane TNFα (tmTNFα) blocks TNF receptor 1 (TNFR1) binding to soluble TNFα, thereby counteracting NF-κB activation. Furthermore, TNFR1 membrane distribution and activation as such depends on S-palmitoylation. Finally, few studies defined that plasma membrane expression of the G protein-coupled receptor (GPCR) CCR5 relies on zDHHC3-, zDHHC7-, and zDHHC15-mediated palmitoylation. In addition, intracellular signaling molecules required for CCR5 signaling and chemotaxis, such as (regulators of) G_α_ proteins and Rac1, are susceptible to palmitoylation. Figure created with BioRender.com (accessed on 9 January 2022).

Diverse studies indicate that S-palmitoylation controls TLR signal transduction. A palmitoyl screen using the RAW264.7 macrophage cell line showed that exposure to lipopolysaccharide (LPS), a prototypical TLR4 ligand found in the membrane of most Gram-negative bacteria, profoundly changes the palmitoyl profile of these cells [36]. Among other proteins, LPS was found to trigger S-palmitoylation and activation of enzymes of the phosphatidylinositol cycle, such as type II phosphatidylinositol 4-kinase (PI4KII) β, leading to the inflammatory activation of macrophages. A recent study further demonstrated that zDHHC6 is involved in S-palmitoylation of myeloid differentiation primary response 88 (MYD88) in myeloid cells, a common adapter molecule in the TLR family [69]. Accordingly, blocking MYD88 S-palmitoylation suppressed TLR-induced inflammation. Of interest, the authors provide evidence that CD36-mediated exogenous fatty acid incorporation maintains the intracellular palmitate pool and is essential for MYD88 S-palmitoylation. Given that CD36 relies on S-palmitoylation for proper function as well, these findings stress the complexity of the CD36-TLR signaling axis and the essential role that S-palmitoylation plays herein. In addition to controlling intracellular TLR signaling, a transmembrane domain-proximal cysteine residue in TLR2 was recently found to be susceptible to S-palmitoylation in dendritic cells [70]. Inhibition of TLR2 S-palmitoylation pharmacologically using 2-bromopalmitic acid (2BP) or by cysteine mutagenesis led to decreased plasma membrane expression and a reduced inflammatory response upon TLR2 ligation. Finally, in contrast to the abovementioned studies, S-palmitoylation can also negatively regulate TLR signaling by promoting lipid raft localization of the Src family member Lyn (Figure 3) [71]. This negative feedback regulation may limit excessive inflammation in response to multiple waves of pathogenic stimuli and prevent septic shock. Collectively, these findings indicate that S-palmitoylation dynamically fine-tunes TLR signaling and plasma membrane expression, thereby closely monitoring the anti-microbial macrophage response.

### 3.3. NOD-like Receptor Signaling

The nucleotide-binding oligomerization domain (NOD) proteins, NOD1 and NOD2, are intracellular receptors responsible for the recognition of cytosolic bacterial peptidoglycans. While NOD1 is ubiquitously expressed, NOD2 is primarily present in immune cells, such as macrophages and dendritic cells [72]. In steady state, NOD1/2 are primarily present in the cytosol, with only a small fraction being associated with plasma and endosomal membranes for the surveillance of bacterial components. Increasing evidence suggests that membrane localization of NOD1/2 is essential for bacterial sensing, nuclear factor-κB (NF-κB) and mitogen-activated protein kinase (MAPK) activation, and the release of inflammatory and anti-microbial mediators [73,74,75,76]. Accordingly, impaired membrane localization of NOD1/2 is associated with inflammatory conditions, such as Crohn’s disease [76]. Given the lack of clearly defined membrane-targeting domains, NOD1/2 were classically considered to be anchored to membranes through cytoskeletal components as well as the plasma membrane and endosomal proteins [77]. However, increasing evidence indicates that S-palmitoylation drives the recruitment of NOD1/2 to bacteria-containing endosomes and other intracellular membranes.

In an elegant series of experiments, Lu et al. recently showed that S-palmitoylation is required for NOD1/2 membrane association and its ability to activate the NF-κB and MAPK signaling pathways in macrophages [77]. Site-directed mutagenesis experiments showed that multiple cysteine residues in NOD1 (Cys558, Cys567, and Cys952) and NOD2 (Cys395 and Cys1033) are S-palmitoylated. In addition, it was demonstrated that S-palmitoylation-deficient mutants of NOD1 and NOD2 are mislocalized and lose their ability to induce NF-κB and MAPK signaling in response to NOD ligands. Additionally, gain- and loss-of-function experiments demonstrated that zDHHC5 is required for S-palmitoylation and the function of NOD1 and NOD2. Finally, the authors provide evidence that several NOD2 polymorphisms associated with Crohn’s disease lead to aberrant S-palmitoylation. The majority of Crohn’s disease-associated NOD2 mutant proteins displayed a 70% to 90% reduction in S-palmitoylation levels and a dysregulated intracellular localization of NOD2. Conversely, a gain-of-function NOD2 variant exhibited enhanced S-palmitoylation and NF-κB hyperactivation, which could be counterbalanced using 2-BP (Figure 3). While these findings stress the importance of S-palmitoylation in NOD2 palmitoylation and the pathology of Crohn’s disease, it is noteworthy to mention that the vast majority of polymorphisms did not involve changes in cysteine residues, and none of them matched the cysteine residues described above. Given that single-nucleotide polymorphisms can generate alternative protein isoforms with divergent structures, it would be of interest to study if altered accessibility of Cys395 and Cys1033 mediates aberrant S-palmitoylation in the Crohn’s disease-associated NOD2 mutants that were experimentally addressed.

### 3.4. Cytokine Receptor Signaling

Cytokines are chemical cues that transmit intercellular signals and retain immune homeostasis. Macrophages are major producers of cytokines and express a plethora of cytokine receptors, which alter their physiology upon ligation. Direct and indirect evidence indicates that cytokines and cytokine receptors undergo S-palmitoylation, thereby affecting cytokine signaling networks and the functional phenotype of macrophages. For example, both the interferon alpha and beta receptor subunits 1 and 2 (IFNAR1 and IFNAR2), which bind to type I IFNs and are highly expressed by macrophages [78,79], are subjected to S-palmitoylation [80]. Counterintuitively, while IFNAR1 S-palmitoylation at Cys463 is essential for phosphorylation and activation of signal transducer and activator of transcription 1 and 2 (STAT1/2), key proteins needed for relaying the transcriptional activity of IFNs, it does not impact IFNAR1 endocytosis, intracellular distribution, or stability at the cell surface (Figure 3) [80]. To date, the impact of IFNAR S-palmitoylation on anti-viral and -tumor activities of IFNs remains unexplored. Vice versa, it remains unclear whether viruses or tumors can hijack IFNAR1 S-palmitoylation to support their replication and growth, respectively.

Similar to type 1 IFN signaling, ample evidence points toward S-palmitoylation having a broad range of functions in the tumor necrosis factor alpha (TNFα) signaling pathway. Transmembrane TNFα (tmTNFα), but not soluble TNFα (sTNFα), was recently found to be palmitoylated at Cys47, thereby promoting lipid raft integration in macrophages and other cell types [81,82,83]. While tmTNFα S-palmitoylation did not affect cleavage of its extracellular domain and consequent TNFα shedding, it diminished TNF receptor 1 signaling by reducing its binding to sTNF [83]. These findings point toward an auto-inhibitory process that likely plays a vital role in the resolution of inflammation. Furthermore, by using human monocytic U937 cells, TNF receptor 1 was recently demonstrated to be constitutively palmitoylated and de-palmitoylated upon ligand binding ^75^. Mutation of Cys248 counteracted TNF receptor 1 S-palmitoylation and interfered with its receptor localization to the plasma membrane and proper signal transduction. In addition, the palmitoyl thioesterase APT2 controlled TNF receptor 1 de-palmitoylation and, thereby, TNF-induced NF-κB activation (Figure 3) [84].

Finally, S-palmitoylation affects interleukin-6 (IL-6) signaling, a pleiotropic signaling pathway that not only regulates inflammation but also affects haematopoiesis, metabolism, and organ development. Collura et al. showed that gp130, a subunit of the IL-6 receptor complex, is palmitoylated by both zDHHC5 and zDHHC8 [85]. Gp130 S-palmitoylation was found to affect its surface localization and downstream STAT3 phosphorylation (Figure 3). As this study was performed in dorsal root ganglion (DRG) neurons, future studies should confirm gp130 S-palmitoylation in macrophages and define its impact on macrophage physiology and inflammation. Altogether, these studies strongly suggest that S-palmitoylation plays a key role in cytokine signaling pathways in macrophages. However, to what extent faulty S-palmitoylation impacts inflammatory disorders or whether S-palmitoylation can be targeted to suppress inflammation remains to be clarified.

### 3.5. Chemotaxis

Chemotaxis is the directional movement of cells in response to a chemical stimulus gradient. Macrophage chemotaxis is crucial during both onset and resolution of inflammation and relies on the expression of G protein-coupled receptors (GPCRs) that bind chemoattractants. One such receptor is the chemokine (C-C motif) receptor type 5 (CCR5), which is highly expressed on immune cells, macrophages in particular. Notably, alongside enhancing chemotaxis, CCR5 acts as an essential co-receptor for macrophage tropic human immunodeficiency virus (M-tropic HIV) entry into target cells, such as CD4^+^ T-lymphocytes and macrophages [86,87]. Mutagenesis experiments, applying single or combined cysteine residue substitutions, showed that CCR5 is palmitoylated in its carboxyl-terminal domain at three cysteine residues (Cys321, Cys323, and Cys324) [88,89], likely catalyzed by the action of zDHHC3, zDHHC7, and zDHHC15 [90]. The absence of receptor S-palmitoylation led to the sequestration of CCR5 in intracellular biosynthetic compartments and a profound decrease in plasma membrane expression [88,89]. However, aside from reduced surface expression and signaling activity, mutants that escaped degradation and reached the cell surface did not display impaired chemokine binding and receptor internalization in response to ligands, such as RANTES, MIP1α, and HIV envelope proteins [88,89]. Alongside CCR5, intracellular signaling molecules required for GPCR signaling and chemotaxis, such as (regulators of) G_α_ proteins and Rac1, are reported to be palmitoylated [91,92,93,94,95]. Importantly, palmitoylation-deficient mutants displayed an altered cellular protein distribution, impaired GPCR signaling, and migratory defects (Figure 3) [91,92,93,94,95]. These studies indicate that S-palmitoylation is a hub and driver of GPCR signaling and function. However, whether the abovementioned functions can be generalized to primary macrophages and to what extent they can be therapeutically exploited to reduce HIV viral load and the pathogenic accumulation of macrophages in inflammatory disorders remains to be determined.

### 3.6. Lysosomal Hydrolase Sorting

Lysosomes are acidified organelles that carry out degradative metabolism essential to many endocytic, phagocytic, and autophagic processes. Proper lysosomal degradation relies on a collection of over 60 divergent hydrolytic enzymes. Mannose 6-phosphate receptors (M6PRs) shuttle between the trans-Golgi network (TGN) and endosomes, and by doing so, deliver newly synthesized acid hydrolases to lysosomes. To date, two distinct M6PRs have been identified, the cation-dependent (CD-M6PR) and cation-independent M6PR (CI-M6PR), that differ in their transport efficacy [96,97,98,99,100].

Few studies indicate that S-palmitoylation of M6PR is necessary for efficient lysosomal sorting. Mutation of Cys34 to alanine markedly reduces M6PR S-palmitoylation, leading to a gradual accumulation of CD-M6PR in lysosomes and total loss of hydrolase sorting function in the Golgi apparatus [101,102]. In another study, McCormick et al. present evidence that, in addition to CD-M6PR, CI-M6PR is S-palmitoylated and that this modification is necessary for efficient retrograde trafficking [103]. The authors further identified zDHHC15 as the acyltransferase responsible for CI-M6PR S-palmitoylation (Figure 3). Noteworthy, given that the abovementioned studies used fibroblast and epithelial cell lines to study M6PR S-palmitoylation, it is difficult to predict whether S-palmitoylation fulfills a similar role in M6PR shuttling in macrophages. However, in support of a role for M6PR S-palmitoylation in macrophages, proteomic profiling identified M6PR S-palmitoylation in membrane fractions of RAW 264.7 macrophages [15]. Importantly, macrophages are well-known for their endocytic and autophagic capacity, continuously scavenging and degrading apoptotic cells, lipids, and pathogens, among many other biomolecules, in their lysosomes. In addition, it is of high interest to study the therapeutic potential of M6PR S-palmitoylation in lysosomal storage diseases as well as disorders associated with the accumulation of pathogenic lipid-engorged macrophages, such as AD, MS, and non-alcoholic steatohepatitis, and following infections with persistent pathogens such as *Mycobacterium tuberculosis*, *Chlamydia pneumoniae*, and *Toxoplasma gondii* [104]. In these disorders, foamy macrophages often display impaired degradative metabolism and contribute to disease progression (reviewed in [105]).

## 4. Therapeutic Implications

Given the broad variety of (patho)physiological processes in which S-palmitoylation is involved, targeting acyl-transferases and -thioesterases holds great therapeutic promise for a variety of disorders. To date, several inhibitors of palmitoylation and de-palmitoylation have been identified, which all efficiently affect the palmitoylation machinery but also have several off-target effects. For instance, 2-bromopalmitate (2-BP), a general irreversible palmitoylation inhibitor, acts as a potent inhibitor of palmitate incorporation and is widely used in in vitro studies [106]. However, it also inactivates several lipid metabolism-related enzymes, such as triacylglycerol biosynthesis, glycerol-3-phosphate acyltransferase, and fatty acid CoA ligase [107], as well as de-palmitoylating enzymes APT1 and APT2 [108]. Aside from 2-BP, cerulenin, tunicamycin, and 2-(2-hydroxy-5-nitro-benzylidene)-benzo[b]thiophen-3-one, also known as compound V, are reported to inhibit palmitoylation [109,110,111,112,113]. Similar to 2-BP, the mode of action of cerulenin and tunicamycin remains largely unclear [109,110,111,112]. Compound V was found to reversible inhibit zDHHC-mediated palmitoylation in vitro by preventing auto-palmitoylation of zDHHC proteins [113]. Finally, several modulators of de-palmitoylation are available, such as palmostatin B and M, ML211, ML348, ML349, and the mitochondrial pan-APT inhibitor MitoFP, which inhibit the divergent protein acyl-transferases [30,107,108,114,115,116,117,118,119]. Yet again, these modulators show off-target effects, and their mode of action remains largely unclear, hampering their clinical use.

Given the limited number of acyl-transferases and -thioesterases, overlapping substrate specificity further complicates the modulation of protein S-palmitoylation. A possible solution for this can be to develop modulators that affect multiple zDHHCs that are involved in the S-palmitoylation of specific target proteins. With respect to the latter, ankyrin repeat domains on the closely related zDHHC13 and zDHHC17 are essential for the interaction with target proteins and could be exploited to modulate S-palmitoylation of specific proteins [120]. Similar, context-dependent changes in expression and divergent cellular and tissular distribution could be taken advantage of to achieve cell type-specific S-palmitoylation of proteins of interest. To add further complexity, acyl-transferases and -thioesterases are subjected to enzyme-mediated palmitoylation and de-palmitoylation themselves. For example, zDHHC16 function and distribution rely on dynamic (de-)palmitoylation by zDHHC6 and APT2 [121]. All in all, overlapping substrate specificity and enzyme-mediated zDHHC S-palmitoylation have hampered the development of clinically relevant modulators of S-palmitoylation.

Besides using enzyme modulators, dietary palmitate impacts the palmitoylation status of proteins. For instance, a palm oil-rich diet leads to accumulation of palmitate in membrane-enriched fractions of murine livers, as well as altered S-palmitoylation of proteins involved in the metabolism of fatty acids and cholesterol, such as fatty acid synthase, fatty acid elongase 2, stearoyl-CoA desaturase-1, CD36, and lanosterol synthase [122]. Similarly, several studies found that a high-fat diet, mainly enriched in animal fats, alters the S-palmitoylation of numerous proteins in peripheral organs and the central nervous systems, including but not limited to CD36 and the GluA1 subunit of the AMPA receptor [46,123,124,125]. These data strongly suggest that dietary palmitate impacts cellular S-palmitoylation, which could be therapeutically harnessed to impact systemic and brain disorders.

Emerging evidence indicates that S-palmitoylation can also be exploited to improve the cellular delivery of drugs and vaccines. In the last decades, researchers investigated potential modifications of liposomes using proteins and glycolipids, among others, to increase the cellular specificity and efficacy of liposomal cargos [126,127]. Recently, it was demonstrated that tuftsin, a hydrophilic Thr-Lys-Pro-Arg tetrapeptide, has the ability to potently bind to receptors on macrophages, monocytes, and neutrophils, thereby driving FcR-mediated phagocytosis and making it a promising tag to promote liposomal cell-specific targeting [127,128,129,130]. However, due to the hydrophilic properties of tuftsin, the addition of palmitate as an anchor arm is needed to allow its binding to liposomes, demonstrating the importance of S-palmitoylation in drug and vaccine delivery in relation to macrophage-based diseases [128,129].

To summarize, protein S-palmitoylation controls macrophage function in health and disease through modulating processes such as endocytosis, intracellular signaling pathways, lysosomal hydrolase sorting, and chemotaxis. In the upcoming years, more research is warranted to fully understand palmitoylation and de-palmitoylation dynamics in macrophages and to assess the therapeutic potential of targeting S-palmitoylation in immunopathologies.
